# Predictors of sunscreen use in United States high‐school students

**DOI:** 10.1002/ski2.370

**Published:** 2024-03-11

**Authors:** Carly R. Stevens, Maxwell Green, Kimberly Hinh, Sofia Chaudhry

**Affiliations:** ^1^ Tulane University School of Medicine New Orleans Louisiana USA; ^2^ Department of Dermatology Saint Louis University School of Medicine St. Louis Missouri USA

Dear Editor,

Skin cancer is the most common malignancy experienced worldwide with the incidence of melanoma increasing yearly.^1^ Although rates of skin cancer are higher among Caucasians, skin cancer in people of colour is often found at later stages and is associated with higher mortality.^2^ Therefore, prevention through education and behaviour modification is of the utmost importance, with the American Academy of Dermatology recommending the use of daily sunscreen to all individuals.

Sunscreen helps protect the skin from the harmful effects of ultraviolet (UV) radiation using UV absorbers. Without this protection, UV damage can induce epidermal and dermal DNA mutations, leading to skin cancer development.^1^ Sunscreen has been shown to reduce of the risk of both melanoma and non‐melanoma skin cancers.^3^ Therefore, prevention campaigns are essential to educating the public on the importance of sunscreen use. Programs such as Sun Protection Outreach Teaching by Students (SPOTS), which teaches school children about the importance of sun protection, are crucial given that an estimated 40%–50% of UV damage is obtained by the age of twenty.^4^, ^5^ The goal of this review is to summarise predictors of sunscreen use among high‐school adolescents in the United States (U.S.).

The Preferred Reporting Items for Systematic Reviews and Meta‐Analysis were used to conduct this review. A comprehensive literature search was done using PubMed, Embase, and Web of Science in November of 2021 using the terms (‘sunscreen’ or ‘spf’ or ‘sun protection’) and (‘high school’ or ‘teen’ or ‘teenager’ or ‘adolescent’). Non‐English articles and those without full‐text were excluded. Articles were included if they met the following criteria: (1) provided quantitative data on predictors of sunscreen use (2) adolescent population (3) conducted in the U.S.

The data was collected by three researchers (C.S., M.G., and K.H.). Initial screening of articles was done using title and abstracts. The final screen was done using full‐text to determine which articles met all inclusion criteria. Any discrepancies were settled by an outside researcher (S.C.). The quantitative data collected was on predictors of sunscreen use in U.S. high‐school students.

The initial database search returned 468 articles. Title and abstract screening yielded 108 relevant articles which underwent full‐text review. Twenty studies were included for qualitative review (Table 1). Studies examining gender showed increased sunscreen use in females compared to males (*n* = 11). Increased sunscreen usage was found in younger adolescents compared to older adolescents (*n* = 4). Caucasian students were more likely to use sunscreen compared to other ethnicities (*n* = 4). Studies also showed increased sunscreen use in populations that believed that were more susceptible to sun damage (*n* = 4). Higher rates of sunscreen use were correlated with higher levels of perceived self‐efficacy, as well as social and familial use of sunscreen (*n* = 2, *n* = 4).

**TABLE 1:** Results of the 20 studies that met inclusion criteria from Preferred Reporting Items for Systematic Reviews and Meta‐Analysis guidelines.

Title	Author(s)	Year	Data	Factors
Prevalence and correlates of sunscreen use among US high school students	Hall, H.I., Sherry, E.J., Saraiya, M.	2001	***N* = 15 399; M = 7445 F = 7828; W = 5407 B = 4283 H = 4106**	Sex, ethnicity, and age
			13.3% (95% confidence interval, ±1.3) of students used sunscreen always or most of the time	
			1. F used sunscreen more than M (18.1%, ±1.9), (8.6%, ±1.2)	
			2. White students used sunscreen more than Hispanic or Black students (16.5%, ±1.9), (10.8%, ±2.8), (4.8%, ±1.7)	
			3. Students aged 14 years or younger used sunscreen (17.5%, ±3.9) more than those aged 15 and older (13.7%, ±2.2), (11.9%, ±2.5), (11.6%, ±2.8).	
Correlates of sunscreen use among high school students: a cross‐sectional survey	Heckman, C.J., Coups, E.J.	2011	***N* = 242**	Sex, sun sensitivity, beliefs, self‐efficacy, and external support
			Sunscreen use was more common in the following populations:	
			1. Females (*p* = 0.004)	
			2. Students with a fairer skin type (*p* < 0.001)	
			3. Students who believed the benefits of sunscreen (*p* < 0.001)	
			4. Students with stronger skin protective norms (*p* < 0.001)	
			5. Students with a higher self‐ efficacy (*p* < 0.001)	
			6. Students whose parent encouraged sunscreen use (*p* = 0.018)	
Sun exposure and sun‐protection behaviors and attitudes among U.S. youth, 11 to 18 years of age	Cokkinides VE, Weinstock M, O’Connell MC, et al.	2001	***N* = 1192**	Sex, age, and sun sensitivity
			Sun protective behaviours were:	
			1. Less likely in M. (95% CI 0.2, 0.5)	
			2. 2.5 more likely in younger students (95% CI 1.5, 4.1)	
			3. 2.6 times more likely in high sun‐sensitivity index skin (95% CI 1.4, 4.9)	
Sun protection practices in preadolescents and adolescents: a school‐based survey of almost 25,000 Connecticut schoolchildren	Coogan PF, Geller A, Adams M, Benjes LS, et al.	2001	***N* = 24 645**	Sex and age
			1. Sun protection practiced were more common in females (35.8%) versus (26.7%)	
			2. Age also influenced habits	
Trends in sunscreen use among US middle and high school students, 2007–2019	Rajagopal G, Talluri R, Chuy VS, Cheng AL, et al.	2021	***N* = 13 513–16 410**	Sex, age, and ethnicity
			1. Females were (92.7%) more likely than males to use sunscreen	
			2. Sunscreen usage decreased by (5%) with each 1‐year increase in student's age	
			3. Sunscreen trends in students by ethnicity: American Indian/Alaskan Native versus H OR = 0.996, Asian versus Hispanic/Latino OR = 1.81, Black versus H OR = 0.403, NWPI versus H OR = 1.117, and White versus H OR = 1.97	
Sunscreen use among US high school students, 1999–2003	Jones SE, Saraiya M.	2006	***N* = 13 601–15,349 M = 51.4% F = 48.6% W = 61.4% H = 16.6% B = 13.9% Other = 8.2%**	Sex, ethnicity/race, and geographic location
			1. Significantly more females (17.7%; 95% CI ±2.0) than males (10.9%; 95% CI ±1.6) routinely used sunscreen (*p* < 0.01)	
			2. More white (17.3%, 95% CI ±1.7) than black (4.4%; 95%CI ±1.3) and Hispanic (11.2%; 95% CI ±1.6) students regularly used sunscreen	
			3. Significantly fewer students in Midwest (OR = 0.7) south (OR = 0.6), and West (OR = 0.8) reported sunscreen use as compared to those in the Northeast (OR = 1.0)	
A characterization of sun protection attitudes and behaviors among children and adolescents in the United States	Patel AR, Zaslow TL, Wren TAL, Daoud AK, et al.	2019	***N* = 860 M = 48% F = 52%**	Sex and age
			1. F reported greater frequency of using sunscreen (*p* < 0.001)	
			2. Age was inversely associated with sunscreen use frequency (*p* < 0.0001)	
Use of sunscreen, sunburning rates, and tanning bed use among more than 10,000 US children and adolescents	Geller AC, Colditz G, Oliveria S, emmons K, et al.	2002	***N* = 10 079 M = 41% F = 59%**	Sex and sun sensitivity
			1. Girls used sunscreen more routinely than boys (40% vs. 26.3%: OR = 1.86; 95% CI: 1.70–2.03)	
			2. Fair skinned children were more likely than olive‐complected and dark‐complected children to report sunscreen use (49% vs. 29% and 20; OR = 1.49; 95% CI: 1.34–1.65) *p* < 0.001	
Knowledge, attitudes, and behaviors toward skin cancer in Maryland youths	Alberg AJ, Herbst RM, Genkinger JM, Duszynski KR	2002	***N* = 2775 M = 45% F = 55% W = 65**	Sex
			1. Girls had significantly higher knowledge scores (*p* < 0.01), more favourable attitudes (*p* < 0.001), and were significantly more likely to report sunscreen use (*p* < 0.001)	
Attitudes of teenagers toward sun exposure and sunscreen use	Banks BA, Silverman RA, Schwartz RH, Tunnessen WW Jr.	1992	***N* = 220 M = 55% F = 45%**	Sex, external support, and knowledge
			1. F (OR = 4.5; *p* < 0.0001), having a best friend who routinely used sunscreen (OR = 3.0; *p* < 0.001) increased use more than males	
			2. Having parents who insisted on sunscreen use when the teenagers were children (OR = 3.0; *p* < 0.006) increased use	
			3. Knowing the maximum time for safe exposure to the sun (OR = 6.2; *p* < 0.0001) was associated with increased sunscreen use	
Changing knowledge and attitudes about skin cancer risk factors in adolescents	Mermelstein RJ, Riesenberg LA.	1992	***N* = 1596 M = 47% F = 53% W = 83% A = 7.6% H = 5% B = 1.1% Other = 3.3%**	Sex and sun sensitivity
			1. F used sunscreen significantly more frequently than males (*X* ^2^ = 51.9, *p* < 0.001)	
			2. High risk skin types used sunscreen more frequently than low‐risk skin types (*X* ^2^ = 19.0, *p* < 0.001)	
Sun Protection Outreach Teaching by Students (SPOTS)‐evaluating the efficacy of skin cancer prevention education for adolescents	Chaudhry SB, Armbrecht ES, Gibbons M, et al.	2021	**N pre survey = 1142 M = 52% F = 48**% Fitzpatrick skin types 1–3, 23% family history of cancer	Knowledge
			**N post survey = 618 M = 47% F = 53%** Fitzpatrick skin types 1–3 83%, 25% family history of cancer	
			1. 26% improvement (*p* < 0.001) in choosing SPF 30+	
			2. 19% (*p* < 0.001) increase in belief that sunscreen can prevent skin cancer	
			3. 61% improvement (*p* < 0.001) in the belief that a tan is unhealthy	
			4. 50% increase (*p* < 0.001) in understanding that tanning is associated with premature ageing of the skin	
			5. 20% improvement (*p* < 0.001) for participants' intent to wear sunscreen often/always during the upcoming summer	
			6. 44% improvement (*p* < 0.001) in those who originally reported to not use sunscreen	
Skin sun‐acne tutorial evaluation among middle‐ and high‐school students in central New Jersey	Irwin B, Mauriello D, Hemminger L, Pappert A, et al.	2007	***N* survey and pretest = 1214, *N* post‐test = 844 M = 52% F = 48%**	Knowledge
			1. All student scores increased by 36.4% (95% CI: 78.8%–81.3%, *p* < 0.001)	
Adapting a skin cancer prevention intervention for multiethnic adolescents	Cassel KD, Tran DA, Murakami‐Akatsuka L, et al.	2018	***N* = 208 A = 51.6% NHPI = 30.4% W = 8.4% H = 3.5% B = 2.7% Other = 3.2%**	Knowledge
			1. Significant increases in student knowledge, attitudes, and intended sun protective behaviours at posttest compared to pretest (*p* < 0.05)	
			2. 12 month follow up survey of subset of students *N* = 42, maintained higher than baseline correct responses in answers about knowledge of the benefits of UVR protection	
A four‐group experiment to improve Western high school students’ sun protection behaviors	Wu, Y. P., Parsons, B. G., Nagelhout, E., et al.	2019	***N* = 1573 M = 50% F = 50% NHW = 62.5%** 29.8% reported family history of skin cancer	Knowledge
			1. Statistically significant (*p* < 0.05) improvements in almost all sun protection behaviours (wearing long pants or long skirt, sunscreen use, and other forms of protective clothing)	
The impact of a skin care and skin cancer prevention lesson on the knowledge and behaviours of high school students	Sharma, J.K., Sharma, S.N.	2020	***N* = 2,688**, return rate of 38.1% (*n* = 1025)	Knowledge
			Post‐lesson on skin cancer prevention:	
			1. 95% CI showed statistically significant change in knowledge gain, knowledge decay, and knowledge persistence	
			2. 12.5% decrease in students reporting wearing sunscreen 0 days a week	
			3. >50% students reported wearing sunscreen 5–7 days a week	
			4. Increase in 9.8% students examining themselves for moles	
Sun‐protective behavior among high‐school and collegiate athletes in Los Angeles, CA	Cohen, P. H., Tsai, H., & Puffer, J. C.	2006	***N* = 996** (612 athletes, 384 controls)	Sex, athlete versus control
			1. F were more likely than male subjects to reapply sunscreen after prolonged sun exposure (*p* = 0.019)	
			2. 12.7% of athletes versus 3.4% of controls (*p* < 0.001) felt that sunscreen impairs athletic performance	
			3. Athletes rated a suntan as more attractive more frequently than controls (*p* = 0.01)	
Predictors of summer sun safety practice intentions among rural high school students	Cho, H., Sands, L. P., & Wilson, K. M	2010	***N* = 219 M = 53% F = 47% W = 95.5%**	Self‐efficacy, external support
			1. Statistically significant increases in predictors of sunscreen among boys in terms of self‐efficacy, response efficacy, and peer norms	
Skin cancer awareness and sun protection behaviors in white Hispanic and white non‐Hispanic high school students in Miami, Florida	Ma, F., Collado‐Mesa, F., Hu, S., & Kirsner, R. S.	2007	***N* = 369 WH = 221 NWH = 148**	Ethnicity
			1. More NWHs than WHs had heard of (37% vs. 19%; *p* < 0.01) and been told how to perform skin self‐examination (11% vs. 3%; *p* < 0.01)	
			2. 19% and 4% of WHs considered their chances to be average or higher than average to develop skin cancer in the future, as compared to NWHs at 26% and 14% (*p* < 0.01)	
			3. 18% of NWH wore sun‐protective clothing most of the time and 6% wore sun‐protective clothing all of the time, compered respectively with 9% and 3% of WHs (*p* < 0.01)	
			4. 28% of NWHs wore sunscreen sometimes, 10% most of the time, and 8% always compared 2*i*th 18%, 8%, and 2% of WHs, respectively (*p* < 0.01)	
Sun‐safe practices in U.S. youth and their parents: role of caregiver on youth sunscreen use	Cokkinides, V. E., Weinstock, M. A., Cardinez, C. J., et al.	2004	***N* = 1192 pairs of youth and parents**	External/parental
			1. Youth whose parents used sunscreen frequently were 30% more likely to frequently use sunscreen (*p* < 0.001)	

*Note*: Bold values indicate surveyed demographics of each individual study.

Abbreviations: A, Asian; B, Black; F, Female; H, Hispanic; M, Male; NHPI, Native Hawaiian Pacific Islander; NWH, Non‐White Hispanic; W, White; WH, White Hispanic.

UVR is the number one preventable risk factor for skin cancer. However, daily sunscreen use is not routinely followed by most individuals. Overall, sunscreen use in adolescents was relatively low, with males, non‐Caucasians, and older adolescents at the greatest risk for not using sunscreen. Males utilised lower levels of sun protection, and they were found to have lower levels of skin cancer knowledge, lower perceptions of being susceptible to skin cancer, and were more likely to view sunscreen as a beauty product rather than a health product.^6^ Non‐Caucasian adolescents and those with less sensitive skin are less likely to believe that spending time in the sun would increase the chances of developing skin cancer, with studies reporting associated risk factors of societal messaging, misinformation, or other social‐cultural norms.^7^, ^8^ Studies analysed demonstrated that older adolescents utilised sunscreen less than younger students, with one study showing a 5% decrease every 1‐year increase in age.^9^ Additionally, adolescent males experience significantly more sun exposure during summer months compared to females.^10^

Skin cancer prevention campaigns can target specific populations in order to generate the most significant impact on changing sunscreen habits. For example, following the SPOTS programme education, one‐third of adolescent participants reported having attempted to increase sunscreen use in a 1‐month post‐programme survey.^4^ With the continued expansion of virtual teaching platforms, more communities are accessible by skin cancer prevention campaigns. Overall, this review shows the need for enhanced sun protection education that can better reach males, non‐Caucasian students, and older adolescents.

## CONFLICT OF INTEREST STATEMENT

The authors declare no conflicts of interest.

## AUTHOR CONTRIBUTIONS

**Carly R. Stevens**: Conceptualization (lead); investigation (lead); writing—original draft (lead). **Maxwell Green**: Conceptualization (supporting); investigation (supporting); methodology (lead); writing—original draft (supporting); writing—review and editing (equal). **Kimberly Hinh**: Conceptualization (supporting); investigation (supporting); writing—review and editing (supporting). **Sofia Chaudhry**: Project administration (equal); supervision (lead); writing—review and editing (supporting).

## FUNDING INFORMATION

This article received no specific grant from any funding agency in the public, commercial, or not‐for‐profit sectors.

## ETHICS STATEMENT

Not applicable.

## Data Availability

Data sharing not applicable to this article as no datasets were generated or analysed during the current study.

## References

[1] Ahmed B , Qadir MI , Ghafoor S . Malignant melanoma: skin cancer‐diagnosis, prevention, and treatment. Crit Rev Eukaryot Gene Expr. 2020;30(4):291–297. 10.1615/critreveukaryotgeneexpr.2020028454 32894659

[2] Brunsgaard EK , Wu YP , Grossman D . Melanoma in skin of color: Part I. Epidemiology and clinical presentation. J Am Acad Dermatol. 2023;89(3):445–456. 10.1016/j.jaad.2022.04.056 35533771

[3] Perez M , Abisaad JA , Rojas KD , Marchetti MA , Jaimes N . Skin cancer: primary, secondary, and tertiary prevention. Part I. J Am Acad Dermatol. 2022;87(2):255–268. 10.1016/j.jaad.2021.12.066 35176397

[4] Chaudhry SB , Armbrecht ES , Gibbons M , Council ML , Knutson A , Lickerman S . Sun Protection outreach Teaching by Students (SPOTS) – evaluating the efficacy of skin cancer prevention education for adolescents. Dermatol Surg. 2021;47(7):926–930. 10.1097/dss.0000000000003093 34167128

[5] Waldman RA , Grant‐Kels JM . The role of sunscreen in the prevention of cutaneous melanoma and nonmelanoma skin cancer. J Am Acad Dermatol. 2019;80(2):574–576.e1. 10.1016/j.jaad.2018.06.069 30012373

[6] Mermelstein RJ , Riesenberg LA . Changing knowledge and attitudes about skin cancer risk factors in adolescents. Health Psychol. 1992;11(6):371–376. 10.1037//0278-6133.11.6.371 1286656

[7] Patel AR , Zaslow TL , Wren TAL , Daoud AK , Campbell K , Nagle K , et al. A characterization of sun protection attitudes and behaviors among children and adolescents in the United States. Prev Med Rep. 2019;16:100988. 10.1016/j.pmedr.2019.100988 31660287 PMC6807366

[8] Hall HI , Jones SE , Saraiya M . Prevalence and correlates of sunscreen use among US high school students. J Sch Health. 2001;71(9):453–457. 10.1111/j.1746-1561.2001.tb07325.x 11794273

[9] Rajagopal G , Talluri R , Chuy VS , Cheng AL , Dall L . Trends in sunscreen use among US middle and high school students, 2007–2019. Cureus. 2021;13(7):e16468, 10.7759/cureus.16468 34422497 PMC8370854

[10] Reynolds KD , Blaum JM , Jester PM , Weiss H , Soong SJ , Diclemente RJ . Predictors of sun exposure in adolescents in a southeastern U.S. population. J Adolesc Health. 1996;19(6):409–415. 10.1016/s1054-139x(96)00050-x 8969372

